# Scar overlapping suture for treating chronic tendinous mallet finger in children

**DOI:** 10.1186/s13018-019-1106-0

**Published:** 2019-02-26

**Authors:** Lei Zhang, Yu-Ming Zuo, Yong-Xin Huo, Guo-Qiang Wang, Liu Zhang

**Affiliations:** 1 Department of Orthopedic Surgery, Hebei Medical University, Shijiazhuang, 050000 China; 2 Department of Orthopedic Surgery, The Second Hospital of Tangshan, Tangshan, 064000 China; 3 Department of Orthopedic Surgery, The Affiliated Hospital of North China University of Science and Technology, Tangshan, 064000 China

**Keywords:** Children, Mallet finger, Tendon repair

## Abstract

**Purpose:**

To evaluate the effect of scar overlapping suture for treating chronic tendinous mallet finger deformity in children.

**Methods:**

Six patients younger than 18 years were investigated retrospectively. The active extensor lags of the distal interphalangeal joint (DIPJ) were all more than 40°, and the passive ranges of DIPJ motion were normal. They were all treated surgically by scar overlapping suture technique, featuring careful overlapping suture of the extensor scar and temporary transarticular Kirschner wire fixation of the DIPJ.

**Results:**

Average follow-up was 3.1 years (ranging from 2 to 5 years). All patients made significant improvement in DIPJ activity. Three patients achieved full active DIPJ extension, whereas one patient had a 10° extensor lag and two patients had 5° extensor lags. All patients achieved normal active flexion ranges and full passive motion ranges of DIPJ compared with their uninjured side. There was no bone dysplasia, pain, or deformity recurrence.

**Conclusions:**

Scar overlapping suture for treating chronic tendinous mallet finger in children is safe and effective. According to the Crawford criteria, all patients were graded as excellent.

## Introduction

Mallet finger deformity is caused by a loss of continuity of the extensor tendon over the distal interphalangeal joint (DIPJ) or a fracture of the base of distal phalanx [1, 2], which were called “tendinous mallet finger” and “bony mallet finger,” respectively [1, 3]. When splinting cannot correct the deformity or when more than 4 weeks have passed since the injury, the mallet finger is considered chronic [4, 5]. Usually, splinting is the first choice to treat chronic tendinous mallet finger [4], but surgery can be considered when there is an extensor lag over 40° or if there is a functional deficit [6, 7]. Several surgical techniques have been adopted for treating chronic tendinous mallet finger, such as tenodermodesis, central slip tenotomy, oblique retinacular ligament, and even arthrodesis [8–10]. This investigation was conducted to assess another technique: scar overlapping suture for the treatment of chronic tendinous mallet finger in children.

## Methods and materials

Between February 2010 and February 2014, the patients younger than 18 years who had accepted scar overlapping suture surgery were retrospectively analyzed. The inclusion criteria included patients younger than 18 years who suffered from chronic tendinous mallet finger with no fixed deformity. Patients with a fracture (bony mallet finger) or fixed deformity were excluded from this study. Written informed consents were obtained from all participants. Our hospital Ethics Committee approved this study.

Six patients were included in the final study. They were all males, with an average age of 10.2 years (ranging from 4 years to 17 years and 6 months). The original medical assistance was not taken when they were injured the first time. When they went to the hospital, at least 1 month had passed. The active extensor lags of DIPJ were greater than 40°, and the passive ranges of DIPJ motion were normal. The movements of the proximal interphalangeal joint (PIPJ) were all normal. The missing active DIPJ extension was associated with difficulties in daily life and sports, and the appearance of the injured finger was considered unacceptable. All of the patients and their parents were eager to undergo surgery, because they wanted to recover as soon as possible. The mechanisms of injury included crush, slash, or axial load to the fingertip. The dorsal swelling of each patient’s DIPJ all subsided preoperatively. The details of the patients are given in Table 1.

**Table 1:** Patient data

Patient	Sex	Affected digit	Mechanism of Injury	Age at Surgery, Y	Follow-up, Y	Full Active DIPJ extension	Full active DIPJ flexion	Pain	Functional limitations	Nail deformity
1	M	L, ring	Slash	7.6	2.0	Y	Y	None	None	None
2	M	R, ring	Axial load	17.6	2.2	10° lag	Y	None	None	None
3	M	L, index	Axial load	14.2	2.7	5° lag	Y	None	None	None
4	M	R, index	Crush	4.0	5.0	Y	Y	None	None	None
5	M	R, ring	Axial load	13.11	2.9	5° lag	Y	None	None	None
6	M	L, index	Slash	6.3	3.8	Y	Y	None	None	None

*M* male, *R* right, *L* left, *Y* yes

## Surgical technique

**Fig. 1:**
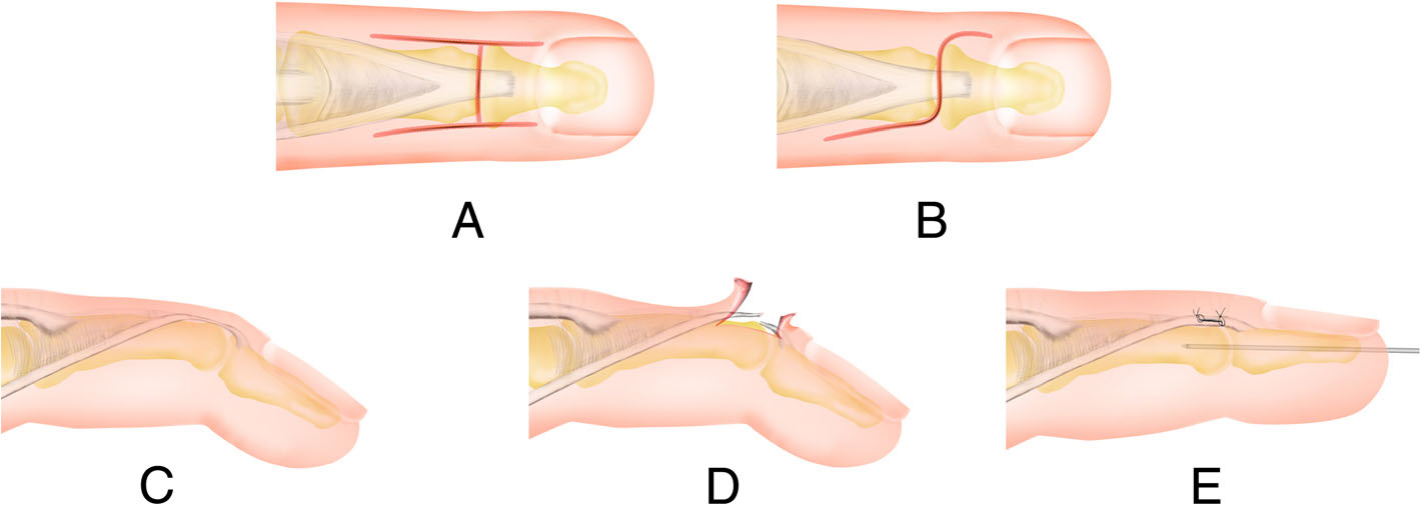
Schematic diagram for the scar overlapping suture technique. **a**, H-shaped dorsal incision was used to approach the terminal tendon. **b**, S-shaped dorsal incision was used to approach the terminal tendon. **c**, The elongated tendon was exposed. **d**, Cut off the elongated tendon. **e**, Overlapping suture was performed to repair the tendon by non-absorb 5–0 Prolene stitches. The DIPJ was fixed 5 degrees hyperextension by Kirschner wire with the diameter of 1.0 mm

**Fig. 2:**
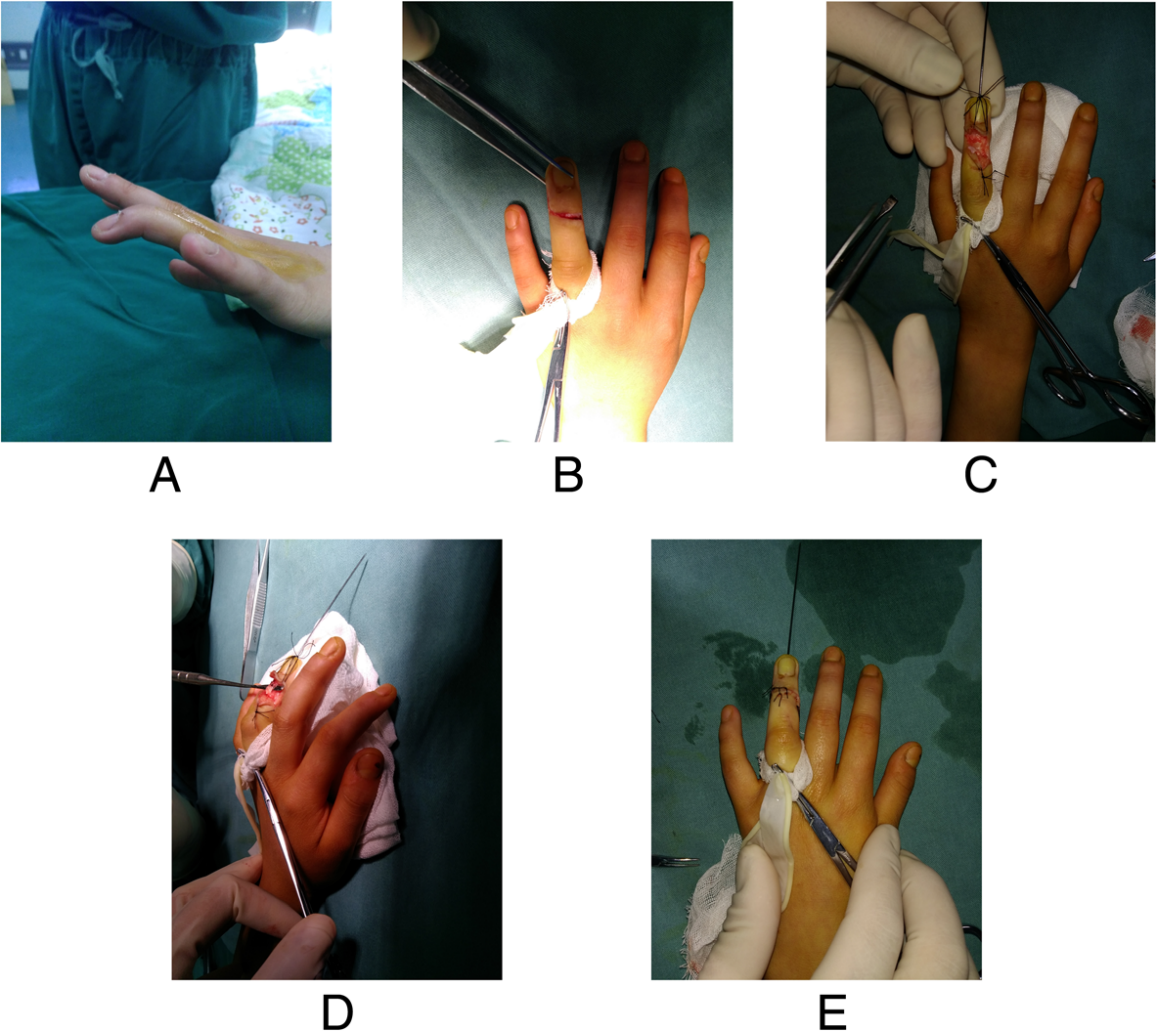
Case 1. A 7.6-year-old boy with a chronic tendinous mallet deformity of the ring finger. **a**, Appearance of the injured finger. **b**, The S-shaped incision over the DIPJ extension crease. **c**, The elongated tendon was sharply cut off. The DIPJ was fixed 5 degrees hyperextension. **d**, Overlapping suture was performed to repair the tendon. **e**, Appearance after wound closure

The operations were performed under block anesthesia or were combined with general anesthesia. An S- or H-shaped incision was made over the dorsum of DIPJ with the transverse limb centered. The skin flaps were carefully raised to preserve the dorsal veins, and then the extensor tendons were exposed. We dissected the aponeurosis carefully to protect the paratenon. Usually, the tendinous scar tissue was difficult to distinguish from the tendon itself, as in chronic mallet finger deformity due to a tendon injury, the tendon healed, lengthened by scar tissue between the edges of the ruptured tendon. We transected the elongated tendon at the site of fibrinous scar tissue and ensured that the tendon cut plane was different from the skin incision plane to reduce the risk of scar adhesions between the tendon and skin. The DIPJ was fixed at 5° hyperextension by Kirschner wire with a diameter of 1.0 mm. Wire placement was confirmed under radiologic examination, and the tip was left outside the fingertip for subsequent removal. We overlapped the proximal and distal tendon stumps without tension (the length of overlapped tendon various with each individual), and interrupted suture was made with the use of nonabsorbable 5-0 Prolene stitches. A palmar forearm plaster splint with wrist neutral and finger extension was used to protect the fix effect. (Figs. 1 and 2) The Kirschner wires and splints were removed 6 weeks after the operations at the same time and subsequently finger exercises were made actively and passively. Assessment was taken, including the movement of DIPJ and PIPJ, pain, limitation in daily life, nail deformity, and the need for reoperation.

## Results

**Fig. 3:**
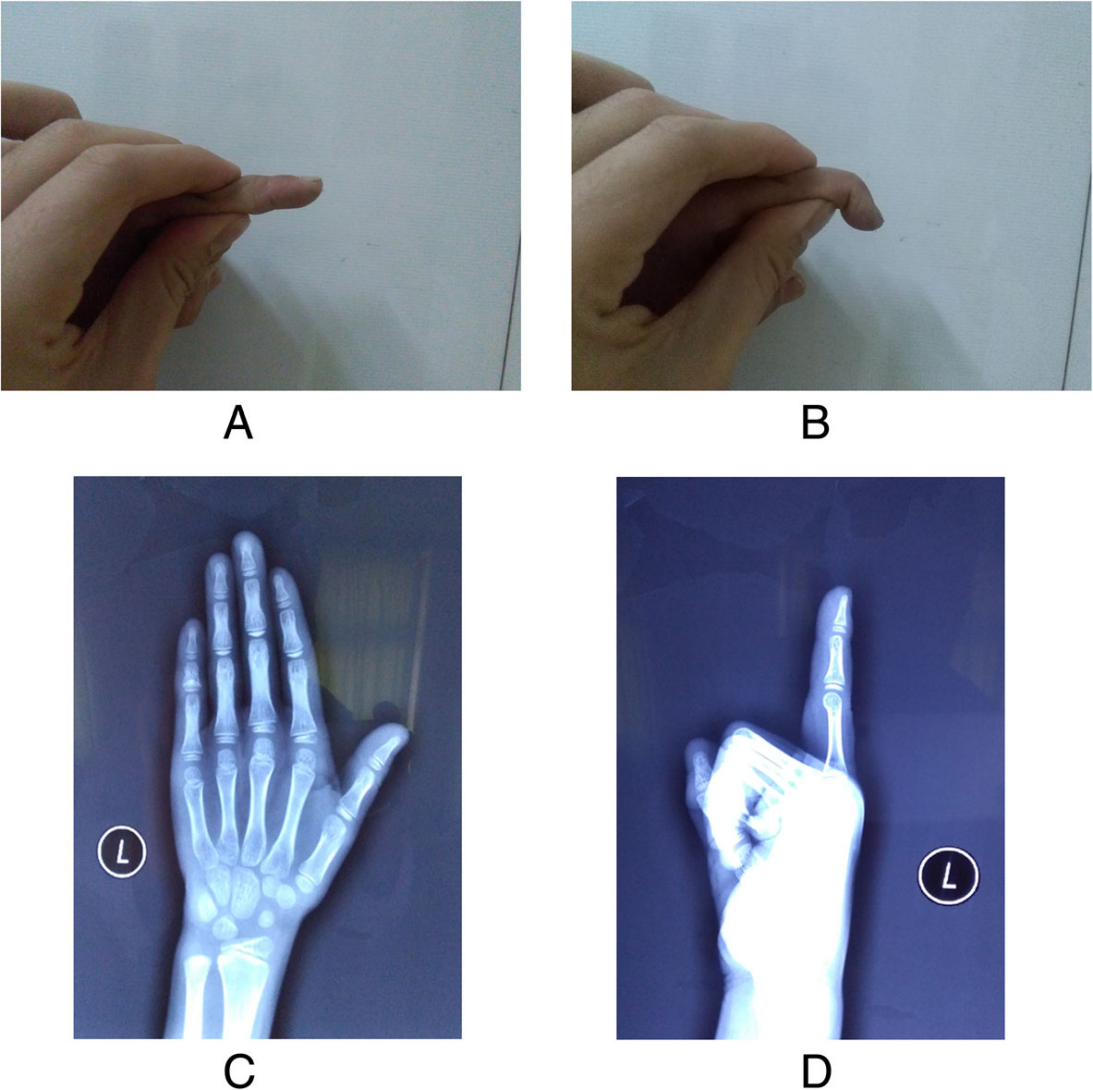
Case 6. **a** and **b**, Photographic view taken at 3.8 years postoperatively, showing the range of DIPJ motion. **c** and **d**, Anteroposterior and lateral radiograph of the injured finger showed no bone dysplasia, and distal phalangeal physis was normal

Average follow-up was 3.1 years (range, 2 to 5 years). At the last follow-up, all patients had significant improvement in DIPJ activity. Three patients achieved full active DIPJ extension, whereas one patient had a 10° extensor lag and two patients had 5° extensor lags. All patients achieved normal active flexion ranges compared with the uninjured side and full passive range of motion of the DIPJ. The active and passive motions of PIPJ were all normal. There was no bone dysplasia, pain, nail deformity, or mallet finger deformity recurrence. All patients reported no limitations using their hands for daily life and sports. According to the Crawford criteria, all patients were graded as excellent [11]. (Fig. 3).

## Discussion

Children with chronic tendinous mallet finger deformity are not commonly seen clinically. A number of challenges exist to treating them. First, it is difficult for young active patients to comply with full-time digital splinting, which results in a higher incidence of failed nonoperative treatment [12]. Second, delay in diagnosis is common, because these injuries often arise from minor trauma and do not always cause immediate functional impairment. Finally, the young patients sometimes do not tell anyone that they have the injuries, and their parents usually ignore the signs of their child’s injured fingers such as fingertip deformity and swelling [13, 14]. As a result, chronic tendinous mallet finger deformity could sometimes be encountered.

Usually, splinting is the first choice to treat chronic tendinous mallet finger [4]; however, because of the children’s nature of activity and poor compliance, the effect of conservative treatment for children is worse than it is for adults [12]. The long period of DIPJ dislocation for disobedient children without splinting can also lead to maldevelopment of the finger, which can eventually result in synarthrosis. Several techniques have been reported for treating a tendinous mallet finger in adults [9, 15–17]. However, for treating children’s chronic tendinous mallet finger deformity, few surgical techniques can be chosen, because the injured finger will still grow over time. Tenodermodesis has been reported as an effective surgical method [6, 7, 18]. However, because of the excision of the dorsal redundant tendon and skin, the dorsal soft tissue of DIPJ could be tight compared with the palmar tissue after the operation. Therefore, the flexion function of DIPJ could be restricted and it can even affect the development of the finger. The scars of the tendon and skin are at the same plane in the tenodermodesis operation, the postoperative adhesions of tendon and skin may get more serious, which can impair the movement of DIPJ. Tenodermodesis technique has these shortcomings. We are the first to describe the scar overlapping suture technique for treating children’s chronic tendinous mallet finger, which can overcome these shortcomings and allow the tension of the elongated tendon to be adjusted and strengthened. These six patients all had an excellent recovery after surgery. This technique differs from previous descriptions. Lind and Hansen [8] and Levante et al. [19] described their surgical techniques for treating adult chronic mallet finger by transecting and resuturing the elongated tendon or shortening–suture of the tendon scar, not scar overlapping suture.

Full passive DIPJ motion and the intact but not swelling dorsal skin are further prerequisites for attempts at surgical reconstruction. Radiographs are also obtained to rule out associated fractures or arthrosis.

All of the six children in this study were males. Three of these boys were injured by axial load to the fingertip, and the injured fingers of all of them were ring fingers. Two of them were injured by slash; neither of them went to a doctor when they were injured, and the wounds were wrapped on their own. One patient who had a 10° extensor lag was 17 years old, and it seems that older patients will not achieve a full-recovery as normal by this operation.

Considering the poor compliance of young patients, we preferred to immobilize the injured finger’s DIPJ with a Kirschner wire with 1.0-mm diameter in children for providing more rigid and more secure immobilization. We used forearm plaster splint to ensure the fix effect preventing the Kirschner wire pulling away or breaking off. We fixed the DIPJ in 5° of hyperextension by Kirschner wire, because more than 5° hypertension might lead to dorsal skin necrosis for the reduced blood in dorsal skin, which eventually causing restriction of the DIPJ flexion. Attention must be paid to the dorsal skin color of DIPJ after fixation, which cannot be pale. No infections or growth disturbances were encountered. After the fixation of the DIPJ by Kirschner wire, we overlapped the proximal and distal tendon stumps to the maximum degree, but without any tension, and then sutured the tendons during operation because too much overlap of the stumps leads to flexion restriction and too little overlap of the stumps results in extensor lag after operation.

One limitation to the current investigation is the relatively small patient population. It is difficult to get a more definite conclusion from limited clinical data, but all of the six patients in this study did have excellent final results without complications. Kardestuncer et al. [6] reported two patients (20%) regained full active DIPJ extension, whereas eight patients (80%) demonstrated a persistent extensor lag of less than 20° by tenodermodesis. The DIPJ motion after scar overlapping suture seems better than that with the tenodermodesis technique.

## Conclusion

This investigation demonstrates that scar overlapping suture for treating chronic tendinous mallet finger in children is safe and effective.

## Abbreviations

DIPJ: distal interphalangeal joint
PIPJ: proximal interphalangeal joint
